# Accelerated Ballast Tank Corrosion Simulation Protocols: A Critical Assessment

**DOI:** 10.3390/ma17102304

**Published:** 2024-05-13

**Authors:** Remke Willemen, Kris De Baere, Rob Baetens, Maarten Van Rossum, Silvia Lenaerts

**Affiliations:** 1 Faculty of Nautical Sciences, Antwerp Maritime Academy, Noordkasteel Oost 6, 2030 Antwerp, Belgium; 2 Department Bioscience Engineering, University of Antwerp, Groenenborgerlaan 171, 2020 Antwerp, Belgium

**Keywords:** ballast tank corrosion, accelerated corrosion testing, simulation protocol assessment

## Abstract

In the realm of accelerated testing within controlled laboratory settings, the fidelity of the service environment assumes paramount importance. It is imperative to replicate real-world conditions while compressing the testing duration to facilitate early evaluations, thereby optimizing time and cost efficiencies. Traditional immersion protocols, reflective solely of full ballast tank conditions, inadequately expedite the corrosion process representative of an average ballast tank environment. Through the integration of immersion with fog/dry conditions, aligning the test protocol more closely with the internal conditions of an average ballast tank, heightened rates of general corrosion are achieved. This augmentation yields an acceleration factor of 7.82 times the standard test duration, under the assumption of a general corrosion rate of 0.4 mm/year for uncoated ballast tank steel, with both sides exposed. Subsequently, the fog/dry test protocol, albeit only resembling the environment of an empty ballast tank, closely trails in terms of acceleration efficacy. The fog/dry test protocol offers cost-effectiveness and replicability compared to the AMACORT CIFD-01 protocol, making it a strong competitor despite the relatively close acceleration factor.

## 1. Introduction

Shipping plays an important role in the logistics chain; the seaworthiness of ships is therefore crucial. Ships are sailing on top of an excellent electrolyte, seawater. This medium tends to be acidic and contains salt which are characteristics accelerating the electrochemical process. They consequently corrode vigorously. Even in the best vessels, well maintained over the years, all steel will in the end try to return to its basic oxidized state. Corrosion equates to material loss, which in turn leads to a reduction in structural rigidity. It is rightfully considered the most significant time-dependent structural degradation factor for ship structures. More specifically, in what follows, we will focus on the water ballast tanks where corrosion plays the most prominent role. These tanks adjust trim, draft and the structural forces on the ship’s hull using seawater to constantly fill up and empty again as a necessary function. Besides this wet/dry effect, many other factors influence the corrosion rate such as coating condition, temperature and stress. These severe in-service conditions make water ballast tanks more vulnerable to corrosion when compared to other areas of the ship [1]. Another reason in promoting the study of the service environment of a ship’s ballast tank is that extending the coating life span of a ballast tank reduces costs for the ship owner [2].

Accelerated corrosion testing is an excellent tool when used as a relative indicator of corrosion resistance [3]. It enhances the durability and reliability of materials and products by evaluating their performance, ultimately reducing the risk of failure due to corrosion. Corrosion is a natural process that causes the deterioration of materials over time, more specifically material loss over time, also expressed as the corrosion rate. This natural process is influenced by environmental factors, such as temperature and humidity. Considering the service environment is consequently of utmost importance when using accelerated testing. Therefore, the more closely the laboratory exposure mirrors the service environment, the more valuable the test data become [3]. It is therefore crucial to clearly define the service environment that the accelerated corrosion testing must replicate in a controlled laboratory setting.

This article specifically addresses ships, with a particular focus on ballast tanks as the service environment. To critically evaluate the related accelerated corrosion testing protocols of this service environment, the key factors influencing the corrosion atmosphere in a ballast tank were defined in Table 1. They were identified as sea water immersion, high humidity and condensation, temperature fluctuations, water motion in the tank and strain following hull loads.

This article investigates the accelerated corrosion testing protocols used to simulate ballast tanks in a controlled laboratory setting and evaluate them with a more near-to-real laboratory test protocol, AMACORT CIFD-01.

We emphasize the importance of realistically simulating the process environment. Seeing that ballast tanks typically remain in good condition for an average period of 18.5 years [2], employing a realistic test simulation could entail a significant waiting period. We need a test environment that correlates with the realistic environment and that shortens the test time so that an early evaluation can be performed. A lengthy test protocol not only involves a prolonged waiting period for results but also incurs higher costs. Acceleration can be the use of continuous salt fog to represent a humid atmosphere, the use of high temperatures or the use of alternating environments like wet and dry conditions. Detailed test protocol information can be found in the existing standards, which are referenced in Table 2.

- The first protocol used in this study is the IMO PSPC_15_ standard, which targets dedicated seawater ballast tanks in all types of ships and double-side skin spaces of bulk carriers [6]. This standard has two main approaches. The first approach based on motion and temperature fluctuations, uses a wave tank chamber. The second is the condensation chamber test (Fog).

Using the wave tank chamber, five service conditions inside a ballast tank should be simulated.

 1. Motion: To simulate a ship’s pitching and rolling, affecting the upper deck condition whereby the panel is subjected to heating and cooling. Cyclically splashing with natural or artificial seawater is used.
 2. Effect of cathodic protection using a sacrificial zinc anode is assessed through cyclic immersion with natural or artificial seawater for two weeks, followed by one week empty.
 3. Motion: To simulate a ship’s pitching and rolling, affecting a cooled bulkhead in a ballast wing tank. Here, splashing is also employed, alongside a temperature gradient of 20 °C, achieved by cooling the reverse side of the plating.
 4. Motion: To simulate a ship’s pitching and rolling, in general using splashing.
 5. Dry: Dry heat is used to simulate boundary plating between heated bunker tanks and ballast tanks in double bottom.

- A commonly known standardization federation is the ISO. The ISO international standard 20340 of 2009 (ISO 20340:2009(E)) [11] provides performance requirements for protective paint systems for offshore and related structures. It aims to age through a cyclic exposure of three stages: UV combined with condensation, salt spray and low temperature exposure of minus 20 °C. Neither the UV exposure nor temperatures of minus 20 °C correlate with conditions inside a ballast tank. For the salt spray test, the ISO refers to their standard 9227 (ISO 9227:2017(E)) [12] which describes corrosion tests in artificial atmospheres and is suitable for checking the preservation of the quality of a metallic material, with or without corrosion protection. In the test method, a neutral 5% sodium chloride solution is atomized under a controlled environment. This fog condition can be related to an empty ballast tank when humid.
- Another international body to deliver standards, test methods, specifications, guides and practices is ASTM, with its headquarters located in America. ASTM D5894 [13] describes a cyclic corrosion/UV exposure of paint on metal using two different cabinets: a cyclic salt fog/dry cabinet and a fluorescent UV/condensation cabinet. Comparable to the ISO standard above, the whole test cannot be correlated with the purpose of our study, only the cyclic fog/dry cabinet can be performed, where a cycle of 1 h fog is combined with 1 h dry-off. Like ISO 9227, ASTM also depicts a salt spray corrosive environment to generate relative corrosion resistance data, as specified in standard B-117 [14]. This standard uses a closed testing chamber in which a salt water (5% NaCl) solution is atomized by means of spray nozzles using pressurized air. When it comes to fog/dry testing, ASTM G 85 [15] is a standard practice for modified salt spray (fog testing) for different purposes of which annex 5 is for diluted electrolyte cyclic fog/dry tests.
- Staying within America, NACE is a knowledge and training center that specializes particularly in corrosion prevention and designs international standards. The two standards of interest are NACE TM0104-2004 [16] and TM0304-2004 [17]. TM0104-2004 is a test method that covers both new construction and maintenance ballast water tank coating systems for tension leg platforms, semi-submersible platforms and floating production and storage offloading systems. It comprises various tests of which the following can be related to our average intern ballast tank condition: Section 8, the seawater immersion test, and Section 13, the hot/wet cycling test using a cyclic salt–fog chamber. TM0304-2004 is a test method of coating systems for the atmospheric zone and splash zone of an offshore platform. Likewise, various tests are considered, among which a seawater immersion test, Section 10, and a test using the program of ASTMD5894 have correlations with this study.
- The Norwegian petroleum industry created a NORSOK M-501 [18] standard inspecting protective coatings applied during the construction and installation of offshore installations and associated facilities. When it comes to the performance requirements of protective paint systems, they refer to ISO 20340.
- In Finland, the Nordtest Method NT Poly 185 [7] describes procedures to evaluate the strain capacity of aged organic coatings on substrates of steel panels, especially of coatings intended for use as protective coatings for ballast tanks. The test procedure is a flexibility test and a cyclic fatigue test where specimens are subjected to strain.

Miwa et al. [19] studied accelerated corrosion testing for evaluating the corrosion resistance of coatings in outdoor environments. He focused on the water absorption and desorption behaviors of anti-corrosion coatings by comparing the wet (humid and fog) time ratio with the dry time ratio of cyclic wet/dry testing. He concluded that when the steel is exposed to a longer period of dryness compared to wetness, this results in lower corrosion rates. Using more wetness will result in higher corrosion rates. When using continuous fog (thus without dry moments), Miwa et al. obtained corrosion rates, which are in line with the cyclic fog/dry exposure with a longer dry time. From this study, we can suggest that using a wet time ration of about 80% can speed up the corrosion rate, as presented in Table 3. Miwa et al. also stipulated that this latter acceleration simultaneously replicates corrosion similar to that experienced in the outdoor environment [19].

## 2. Materials and Methods

### 2.1. Key Factors Included in Accelerated Corrosion Protocols

Not all key factors present in a ballast tank environment were included in our assessment of accelerated corrosion test protocols. Given that an average ballast tank environment is being considered, the following factors were not included:

- M: Considering that motion/sloshing occurs only in partly filled tanks, which is not representative for an average ballast condition, it will not be regarded as a key factor in this evaluation of accelerated corrosion testing protocols;
- S: Considering that stress/strain initially leads to cracking and subsequently to corrosion, we will not consider it as an environmental factor being a first point of contact affecting corrosion;
- CP: Given that in accelerated corrosion testing, cyclic exposures alternate rapidly, the efficacy of sacrificial anodes may be called into question. Therefore, the performance of sacrificial anodes will degrade with cyclic exposure to both empty atmosphere and immersion in seawater, significantly diminishing the efficacy of cathodic protection [10]. De Baere et al. [20] investigated the effectiveness of sacrificial anodes in ballast tanks across more than 100 merchant vessels. The investigation concluded that no significant difference was found in observations regarding corrosion occurrence between ship populations with and without sacrificial anodes, across all ship ages. Much earlier, a report dated 1988 and published in 1991 by the Ship Structure Committee confirmed [21] this suggestion, stating that zinc anodes did not provide adequate protection for uncoated ballast tank surfaces. This study will not consider the use of sacrificial anodes.

In this study, the key factors included are subsequently “I”, “F” and “D”, used to and evaluate existing accelerated corrosion testing protocols. However, to what extent? Verstraelen et al. [22], De Baere et al. [23] and Willemen et al. [2] inspected the ballast tanks of 172 randomly chosen ships between 2007 and 2019. The ships were visited as the opportunity arose. Analyzing the resulting database, it can be inferred that during 85% of those visits, the chief-officer or the officer on duty was queried about the average percentage of time their ballast tanks were filled. The testimonies revealed a value of 47%. We can assert that, on average, a ballast tank is immersed for half of the time. Similar to immersion, it is suggested that a ballast tank is empty for the other half of the time.

In an empty stage, the key factors D and F are vital. The question that arises is how much fog time and how much dry time will have to be considered. Based on Table 3, a cyclic combination of 2/3 F and 1/3 D during the phase when a ballast tank is 50% empty will not only correspond to an average ballast tank internal atmospheric condition (as described previously) but also accelerate a corrosion test protocol. In this case, like Takashi, the tank is in total wet for approximately 80% of the time (immersion + fog).

For existing accelerated corrosion testing protocols, the wet/dry time ratio will be noted. The protocols will be compared based on scores ranging from 0 to 4. The higher the score, the more likely it represents the average ballast tank internal atmospheric condition. The following scores are applicable:

0 = no correspondence;

1 = less than partial correspondence;

2 = partial correspondence;

3 = more than partial correspondence;

4 = good correspondence.

The first protocol used in this comparison is the IMO PSPC_15_ standard. As we only consider the average conditions, which involve either completely full or completely empty ballast tanks, only service condition 5 of this protocol was retained in our study.

### 2.2. Experimental Evaluation of Accelerated Corrosion Testing Protocols

For the experimental study, three accelerated corrosion protocols were chosen based on the theoretical evaluation. First, we assumed a filled ballast tank and thus considered “I” immersion as the environmental parameter. Furthermore, we considered an empty ballast tank where the environmental conditions “F” fog as well as “D” dry took place. The last protocol implied a cyclic combination of both filled and empty ballast tanks, using the cyclic regime of a 50% immersed and a 50% empty ballast tank, representing the average ballast tank environmental conditions. The latter was named AMACORT CIFD-01 (Cyclic Immersion Fog Dry).

Each condition was tested for a total period of 12 weeks (14 periods of 6 days) or 2016 h, as follows:

Condition filled ballast tank “Immersion” (a total of five steel samples were tested).

- This correlates with factor 4 to a filled ballast tank condition.
- The samples are immersed for the total test time in artificial seawater at 40 °C.
- Based on NACE TM0104-2004 and NACE TM0304-2004, the test time and water temperature were set.
- The seawater was made modifying ASTM standard D 1141, the standard practice for the preparation of substitute ocean water, by leaving out the heavy metals.

Condition empty ballast tank “fog/dry” (a total of five steel samples were tested).

- This correlates with factor 4 to an empty ballast tank condition.
- The samples are subjected to a cyclic fog/dry exposure, composed of 4 h fog at 25 °C (2/3 wet) followed by 2 h dry-off at 35 °C (1/3 dry).
- Based on ASTM G85 annex 5, the temperatures were set, implying that the fog was performed at room temperature and the dry-off at an elevated temperature. PSPC_15_ also uses room temperature for the condensation chamber testing. If only fog exposure testing is considered, referring to ASTM B 117 as well as ISO 9227:2017(E), a salt solution is atomized at 35 °C.
- The fog solution was made according to ASTM B 117.
- For the fog/dry test, a certified salt spray cabinet (Q-FOG SSP600; Labomat, Saint Denis Cedex, France) was used.

Condition ballast tank “AMACORT CIFD-01” (a total of thirty steel samples were tested).

- This correlates with factor 4 to a ballast tank condition.
- Both “IMMERSION” and “FOG/DRY” will be alternated every three days, resulting in a cyclic immersion fog/dry exposure.
- After every fog/dry phase, the samples were rinsed with demineralized water not significantly affecting the artificial seawater composition of the immersion test set up.

The immersion test set-up was an artificial seawater-filled basin placed “au bain-marie” where the temperature was regularly monitored. Table 4 indicates that the temperature remains constant at 40–41 °C. In this basin, plastic racks were supporting the steel samples, separating them from each other.

### 2.3. Corrosion Rate Determination

Bare steel coupons of grade A mild of 150 × 100 × 3 mm^3^ were subjected to the three testing protocols over a time frame of 3 months (2016 h). The general corrosion rate was evaluated by regularly measuring the samples weight during testing. At the end, the weight was measured again, before and after all the rust was removed. Rust removal took place in two stages. First, the samples were glass bead blasted to remove the excess rust and then etched for 5 min in Clarke’s solution (20 g/L Sb_2_O_3_ + 60 g/L SnCl_2_ in 23% HCl). Afterwards, the samples were rinsed in demineralized water and dried on absorbent paper.

The corrosion rate was expressed in accordance with ASTM G 31 [24], as follows:

Corrosion rate in mm/year = (K × W)/(A × T × D) (1)

where

K = a constant = 8.76 × 10^4^ for the desired unit mm/year;

T = time of exposure in hours = 2016 h;

A = area in cm^2^ (area of the exposed metal);

here A = (15 cm × 10 cm) − area of the sample label hole = 150 cm^2^ − π 0.25^2^ = 149.80 cm^2^;

W = mass loss in g;

D = density in g/cm^3^ = 7.85 g/cm^3^ (grade A mild steel).

The corrosion rate can also be given in g·m^−2^·day^−1^ as mentioned in Table 3. This can be described as follows:

Corrosion rate in g·m^−2^·day^−1^ = W/(A × T) (2)

where

W = mass loss in g;

A = area in m^2^ (area of the exposed metal) = 0.01498 m^2^;

T = time of exposure in days = 84 days.

As a reference, to compare the laboratory results, a general corrosion rate of 0.4 mm/year for uncoated ballast tank steel with the two sides exposed was used.

### 2.4. Aging Determination

An estimation of the aging period achieved with these accelerated corrosion testing protocols was obtained based on the weight change. The average weight loss can be translated to a % of material loss. Knowing the thickness of the bare steel samples, the material loss % can be translated to an obtained thickness reduction. Assuming an average uncoated steel ballast tank corrosion rate of 0.4 mm/year and if the % in weight loss corresponds to the % in thickness loss, we can approximate the obtained ageing time. The acceleration factor is calculated using the following formula:

Test time × acceleration factor = number of years aged(3)

### 2.5. Visual Assessment of Corrosion Product Development

In addition to the weight loss, corrosion was evaluated through the color of the resulting corrosion in accordance with Klenam et al. [25]. The palette of colors described are yellow, red, brown, black and green. They were linked to our key factors, as illustrated in Table 5 below:

### 2.6. Verification of Sample Preparation

Even though the present study presented in this article is a comparative study where all samples underwent the same pretreatments and after treatments, the effect of etching on the weight loss, as well as the effect of glass bead blasting, was investigated to examine the potential influence in the weight analysis.

A total of 4 grade A mild steel samples (150 × 100 × 3 mm^3^) were glass-bead-blasted and subsequently weighed. They were then etched and weighed again. The same procedure was followed for four different grade A mild steel samples (150 × 100 × 3 mm^3^), but they were first etched, then weighed and subsequently glass-bead-blasted and then weighed again.

## 3. Results

### 3.1. Evaluation of Existing Accelerated Corrosion Testing Protocols

In Table 2, the existing testing protocols are evaluated for simulating an average internal ballast tank atmosphere using the correlation scoring and taking the following assumptions into account:

- only the inside ballast tank atmosphere is considered;
- for 50% of the time, an average ballast tank is filled with seawater (=immersion);
- when empty, it is predominantly humid (2/3 fog or humid exposed);
- high temperatures leading to dry heat atmospheres are not to be excluded inside an empty ballast tank (1/3 dry-off exposed).

We can conclude that the existing accelerated corrosion testing protocols either focus on empty ballast tank conditions or filled ballast tank conditions. The combination, or the total condition, correlation is missing.

### 3.2. Corrosion Rate under the Different Accelerated Test Regimes

Measuring shell plate thickness is probably the single and most important method for establishing the overall condition and rate of deterioration of an iron hull [26]. A reduction in plate thickness means a reduction in weight. This weight reduction is the essence of the experimental evaluation of the existing test protocols, where both (empty and full) conditions are considered.

**Figure 1:**
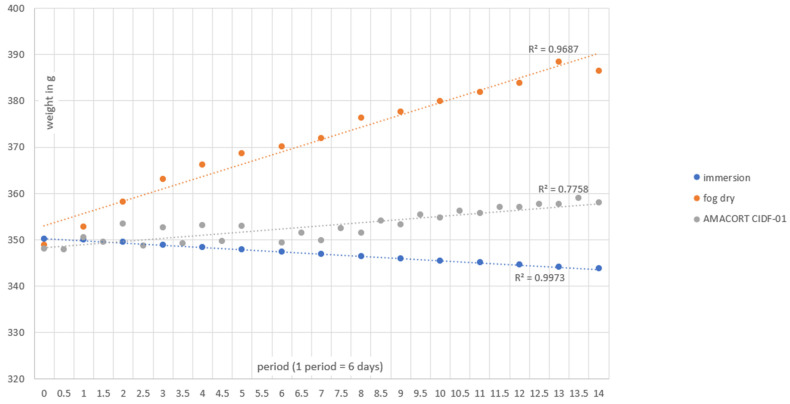
Weight evolution of the accelerated corrosion test protocols: immersion (blue), fog/dry (orange) and AMACORT CIFD-01 (grey).

Over 14 periods of 6 days, the immersed samples reveal that there is a gradual decrease in total weight (Figure 1). The fog/dry samples show a rapid increase in weight due to the rust being formed on the surface. The AMACORT CIFD-01 samples also reveal a gradual increase in weight, but much less than the fog/dry samples likely due to the intermediate immersion where rust could loosen. These trends can be seen in Figure 1 below.

From Table 6, you can see that the variation in the weight change in the AMACORT CIFD-01 test protocol is higher compared to the individual immersion and fog/dry test protocols. Nevertheless, the coefficient of variation (or the ratio of the standard deviation σ to its mean) is lower than one in the three conditions, which can be considered as low.

**Figure 2:**
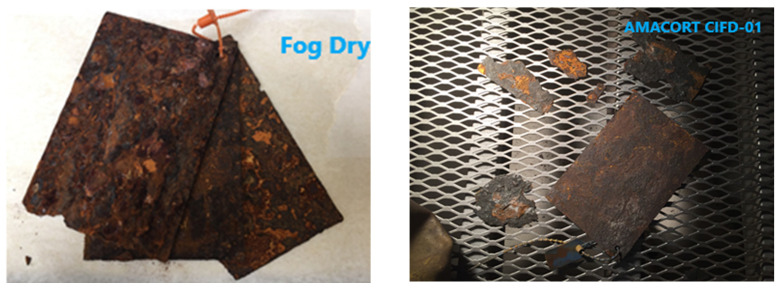
Pattern of loosening rust of the samples after the fog/dry and AMACORT CIFD-01 accelerated corrosion test protocols.

**Figure 3:**
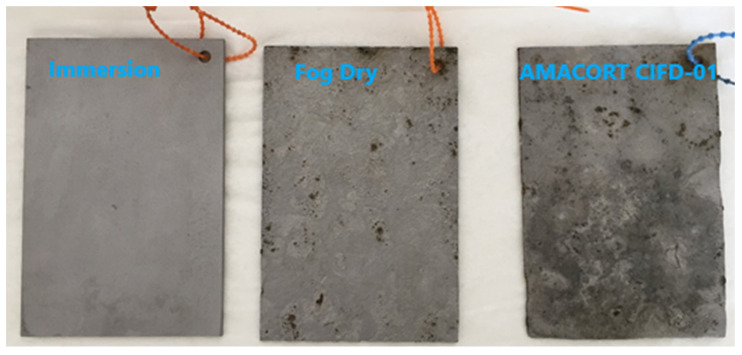
Steel sample look after the accelerated testing and after cleaning.

The fog/dry samples presented rust sheets on either side of the samples that came off easily and the AMACORT CIFD-01 samples presented pieces of big and small rust patches (Figure 2). After rust removal, the latter remaining surface gave a rougher and more degraded surface (Figure 3).

Combining full and empty ballast tank conditions in an accelerated corrosion testing protocol results in higher corrosion rates (Table 7).

Through the determination of the aging period achieved with these accelerated corrosion testing protocols, both the fog/dry and the AMACORT-CIDF-01 protocols resulted in significant acceleration of the corrosion process of the steel, with factors of 6.52 and 7.82, respectively, as presented in Table 8. This renders them both suitable candidates for the accelerated testing of ballast tank corrosion. On the other hand, the immersion test protocol cannot be considered a valid protocol, as the acceleration factor is less than one. The ageing obtained is less than the test time used.

### 3.3. Color Analysis

From the color palette indicated in Table 5, green is associated with buried structures and microbial actions. Guilbaud et al. [27] investigated green rust in aqueous media and stated that the formation of green rust is common in this environment and that the mineral has been found in association with microbial communities and various mineral assemblages. Consequently, we did not expect to detect green rust in our evaluation. The other colors are associated with water exposure, moisture content and oxygen content and thus could be part of the color palette to be detected in our evaluation.

**Figure 4:**
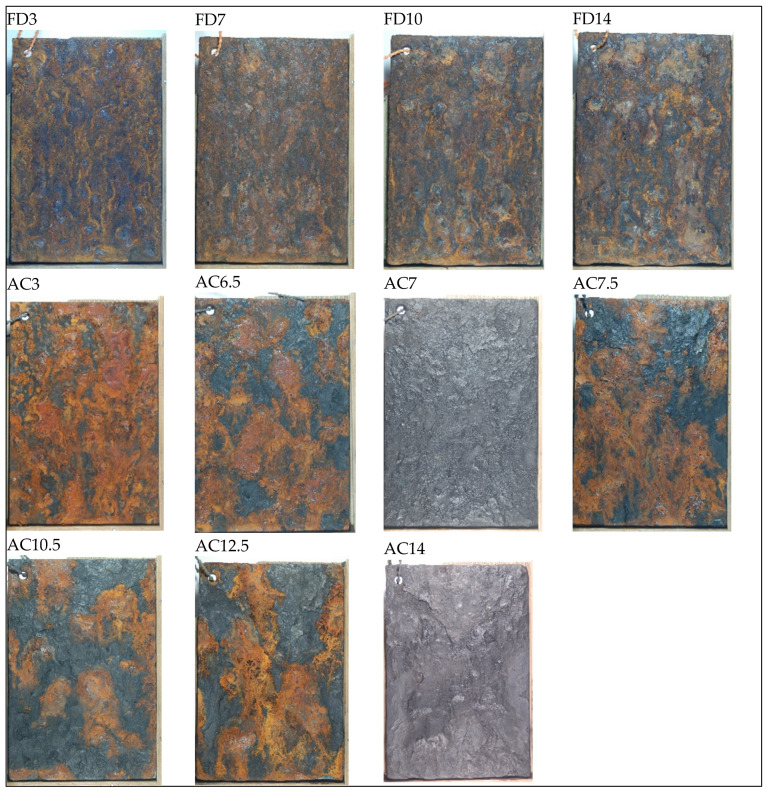
The color palette of the samples during and after the fog/dry (FD pictures) and AMACORT CIFD-01 (AC pictures) accelerated corrosion test protocols. The digit(s) after the indicated FD or AC refers to the number of periods the samples were exposed to the respective acceleration test. Thus, 1 period equals 6 days; 3 stipulates consequently the first mid-half period in the total exposure time; 6.5, 7, 7.5 stipulate halfway in the total exposure time; 10 and 10.5 stipulate the mid second half of the total exposure time; 12.5 nears the end of the total exposure time and finally 14 stipulates the end of the exposure time.

Following the immersion protocol, the entire surface presented gray steel color without traces of brown, black, yellow or red. The latter can also be seen after the immersion phases from the AMACORT CIFD-01 test protocol as presented (Figure 4). Therefore, only the fog/dry and the AMACORT CIFD-01 test protocols were compared for the color analysis.

When subjected solely to a fog/dry atmosphere, the predominant rust color was brown, with some black and yellow, as depicted in Figure 4. In Table 5, brown corresponds to a low moisture content and a high oxygen content, both of which can be correlated with the dry-off condition. Despite the fog/dry test protocol only utilizing 1/3 of the time for dry-off, it appears that this condition is dominantly present in the color palette. Streaks are visible in yellow and black, colors that correspond to a high moisture content or water exposure, respectively. This can be related to the fog dripping on the panels placed under an angle in the Q-FOG following the ASTM B 117 and ISO 9227:2017(E) chamber conditions, namely between 15° and 30° from the vertical.

The AMACORT CIFD-01 protocol shows in Figure 4 a rough surface with lava-like colors and with big red and black stains. Both colors can be linked to water exposure, following Table 5. The pictures in Figure 4, taken after the fog/dry cycle, indicate that the immersion phase has a dominant effect on the color palette. The two pictures where the surface presents a gray appearance were taken after the immersion phase.

### 3.4. Sample Preparation Validation

The average difference in weight using etching was 0.19 g, with a coefficient of variation of 0.31. It indicates a low variance and no predominant difference in weight. Regarding the glass bead blasting, the weight difference is very small, namely 0.02 g, implying that the coefficient of variation is not relevant.

The reason etching might appear to have slightly higher weight difference values compared to the values of the influence of glass bead blasting could be related to the fact that the final weighing occurred immediately after the paper drying from the etching procedure so moisture is not completely eliminated.

## 4. Discussion

Miwa et al. [19] showed more severe values in corrosion rates (expressed in g/m^2^/day), using cyclic humid and fog/dry cycles than the fog/dry cycle used in this study. It should be noted that Miwa used more severe environmental conditions, such as a fog temperature of 35 °C, whereas in this study we used 25 °C. Additionally, Miwa employed a humidity of 50 °C and a drying temperature of 60 °C, whereas we dried off at 35 °C. Higher temperatures inevitably result in higher corrosion rate values. The Materials Technology Institute even states as a rule of thumb that the corrosion rate of a metal doubles for every 10 °C increase in temperature [28].

When it comes to data about the corrosion rates of ballast tank plating, different values are found in the literature. Chemco International [29] mentions for a coated ballast tank a rate of corrosion of about 0.22 mm/year. The crack assessment criteria study of Ajit Nair et al. [30] referred to Beghin [31] who proposed a rate of corrosion for unprotected ballast tanks of 0.4 mm/year and for coated ballast tanks at 0.2 mm/year. Gudze and Melchers [32] conducted an experimental trial using unpainted mild steel coupons exposed inside the seawater ballast tanks of an operational naval ship. With the tank fully ballasted 25% of the time, they ended up with an average corrosion rate of 0.163 mm/year. The IMO stipulates corrosion additions for one side of the coated ballast tank structural member, ranging from 0.1 mm to 0.15 mm/year [33]. In this study, we assumed the worst-case value from the above-mentioned data found after 2000, namely an average uncoated ballast tank corrosion rate of 0.4 mm/year (both sides exposed). This value appears to be the average from the data published in 1991 by the Ship Structure Committee [28]. During the time frame between 1981 and 1982, 32 VLCCS were surveyed using ultrasonic instruments. The publication in 1991 comprises a report dated 1988. It suggests that for two-sided unprotected segregated ballast tank steel, values between 0.3 mm per year and 0.85 mm per year were found in locally high-stress areas.

The AMACORT CIFD-01 set-up needs immersion basins and the Q-FOG. This set-up is consequently more costly. It also demands supplementary manipulation, which means a higher labor cost. The AMACORT CIFD procedure requires considerable effort, as the samples need to be manipulated from the Q-FOG chamber to the immersion basins every 3 days. This effort can be quantified in monetary terms, and it raises the question of whether this effort is justified by the increase in acceleration factor obtained compared to the fog/dry cycle.

In the repetition of the three test protocols by students of the Antwerp Maritime Academy, significant differences in results were observed [34]. When considering repeatability (same team and same set-up), it is essential to note that repeatability conditions entail independent test results obtained with the same method, on identical test items, in the same laboratory, by the same operator, using the same equipment and within short intervals of time [35]. Here, some conditions are violated, such as the same operator as well as the method, since multiple power cuts were experienced during the test, over a long period (in terms of hours). When formulating replicability (different team and same set-up), where measurements are obtained by a different team using the same measurement procedure and the same measuring system, under the same operating conditions, in the same or a different location on multiple trials [36], we still violated the operation conditions with the power cuts experienced. Discontinuities in testing impact the results. ASTM B-117 formulates that short daily interruptions necessary to inspect, rearrange or remove test specimens, to check and replenish the solution in the reservoir and to make necessary recordings are allowed [14]. The cumulative maximum time for these interruptions is held to 60 min or less per day. It is recommended to have only one interruption per day if possible. The latter unforeseen violation in terms of repeatability and replicability could explain the significant difference in outcome. It should also be kept in mind that the immersion and fog/dry protocols implicate exposures to a well-regulated constant atmosphere, whilst the AMACORT CIFD-01 demands manipulations every three days removing the samples manually from one atmosphere to the other (from the basins to the Q-FOG and vice versa). In addition, after the Q-FOG the samples are rinsed with demineralized water before being placed in the basins. Performing it poorly can lead to a different test outcome. We can state that the AMACORT CIFD-01 procedure is very sensitive to reproducibility, where reproducibility according to Plesser implies that measurements can be obtained by a different team, a different measuring system, in a different location on multiple trials [36].

It is stated that the immersion test cannot be considered as an accelerating corrosion test. However, it should be noted that this could be a valid test to investigate the permeability of coatings, which can be linked to coating performance. International Marine Coatings states that highly cross-linked chemically curing systems are likely to have relatively low permeability characteristics and film thickness can affect it. In general, thicker films delay the passage of oxygen and water to the steel surface [37].

In addition to general corrosion, pitting is also present in the ballast tanks. For coated tanks, it is mainly caused by the action of a localized corrosion cell on a steel surface due to the breaking of the coating [38]. As the coating breaks, the subsequent oxidation of the underlying metal in the anodic zones results in the local formation of a pit. Pitting of uncoated tanks is mainly due to the presence of contaminants or impurities on the steel. Shallow but very wide scabby patches are formed, the appearance of which resembles a condition of general corrosion [38]. Gutze and Melchers [32] suggested the following in this respect: “General or ‘uniform’ corrosion as estimated from mass loss experiments is of main interest. It may involve the coalescence of multiple corrosion pits, as evident by visual observation of unprotected mild or low alloy steels as used in the construction of many ships, including naval vessels.” In this study, we examined general corrosion of uncoated steel without the presence of contaminants or impurities.

Many interesting debates can be found about color realism [39,40]. Color perception is subjective, and our perception of physical things involves identifying objects by their appearance, in which colors play a vital role. The color analysis made was found to be an interesting addition by the authors and is undoubtedly worth elaborating on and substantiating with additional analyses.

The primary focus of this study was on utilizing ‘weight loss’ as a benchmark, as during vessel inspections the longevity of steel is assessed through thickness degradation. On-site inspections also involve subjective evaluations by surveyors. We opted for a pragmatic approach aligned with on-board practices and refrained from conducting additional scientific analyses such as SEM-EDX analyses of rust. However, further exploration of this topic would be highly engaging and could provide valuable insights. In future research on accelerated corrosion testing, it would also be beneficial to incorporate open circuit corrosion potential measurements.

## 5. Conclusions

Ballast tanks are filled 50% of the time (wet plates), and during the empty stage, they are mostly very humid, combined with drying moments. A cyclic combination of 2/3 F and 1/3 D in an empty stage would not only accelerate a corrosion test protocol but also correlate with an average ballast tank’s internal atmospheric conditions. Hence, the tank is wet for approximately 80% of the time, considering both immersion and fog conditions.

Existing accelerated corrosion testing protocols either focus on empty ballast tank conditions or filled ballast tank conditions. However, the combination, or the total condition, correlation is lacking. The immersion test correlates with the filled internal average ballast tank environment. This test protocol cannot be considered as an accelerated test protocol as no aging is obtained. The fog/dry test correlates with the empty internal average ballast tank environment. This test protocol accelerates with a factor of 6.52 times the test time. From the color palette observed on the samples after testing, it is evident that dry-off condition is dominantly present, despite the fact that the fog/dry test protocol only uses 1/3 of the time for dry-off. Through a combination of immersion and fog/dry, and thus correlating an internal average ballast tank environment using the AMACORT CIFD-01 protocol, higher corrosion rates are obtained with an acceleration factor of 7.82 times the test time. The color palette on the samples after testing indicates that the immersion phase has a dominant effect. So, the AMACORT CIFD-01 accelerated test protocol is indeed the fastest procedure that also correlates with the realistic environment.

The shorter time needed to obtain results does not necessarily entail lower costs, as the manipulations to be performed are more demanding in the AMACORT CIDF-01 protocol and the set-up installation is more expensive. Given the relatively close acceleration factor of the fog/dry test and the AMACORT CIFD-01 test, and considering that the fog/dry test requires fewer manipulations, the fog/dry test is a strong competitor. Additionally, the fog/dry test holds an advantage due to its replicability.

## Figures and Tables

**Table 1:** The key factors influencing the corrosion atmosphere in a ballast tank.

Key Factor	Code	Description	Principle and Remarks
Immersion	I	Seawater immersion	A ballast tank is filled with seawater to improve stability and stress as well as changing draft and trim. When a vessel is sailing without cargo, she is said to be in ballast. She then has her ballast tanks filled with water (immersed with seawater), ensuring a minimum draught to maintain stability as well as a specified trim to keep the propeller immersed for maneuvering efficiency. Some vessels have permanently filled ballast tanks. Container vessels for instance will seldom operate in a full ballast condition, and consequently they keep some ballast tanks constantly full while leaving others empty.
Fog	F	High humidity and condensation	After emptying a ballast tank, the atmosphere remains highly humid. A ballast tank is an enclosed space with limited natural ventilation. Since it is partly filled with seawater, it can be stated that it is consistently humid most of the time. The latter is supported by the ventilation and dehumidification procedures found in the literature, for instance the work of Appleman [4] who elaborated the ventilation and dehumidification of ship ballast tanks for blasting and coating. A ballast tank is rarely, if ever, 100% empty. It is rare that we can make a ballast tank completely dry. Being a humid atmosphere, when the outside temperature decreases, the steel plate will cool down, leading to condensation. The high humidity, coupled with constant condensation, creates a corrosive environment where the electrochemical conditions necessary for corrosion to occur are present [5].
Dry	D	Temperature fluctuations	When empty, the atmosphere in the tanks can warm up and dry out considerably due to the sun, particularly near the main deck and the upper sections of the ship’s sides, or when located adjacent to a fuel tank or a cargo tank (especially for oil- and chemical tankers). The Performance Standard for Protective Coating regulations [6], PSPC_15_, became mandatory on 1 July 2008 for dedicated seawater ballast tanks on all types of ships of not less than 500 gross tonnage and for double-side skin spaces arranged in bulk carriers of 150 m in length and upwards. The PSPC_15_-approved tests stipulate that dry conditions at 70 °C simulate the region between the heated fuel oil tank and the double bottom. The Nordtest Method NT Poly 185 [7] also notes temperatures of 70 °C and higher for conditions in ballast tanks, namely near the top of the tanks, as a result of the sun’s influence on the decks in tropical areas.
Motion	M	Sloshing or water motion in a tank	Free liquid moving in partially filled tanks causes sloshing motions. As a result, one will avoid partially filled ballast tanks because they directly impact the stability of a vessel, a phenomenon known as the free-surface effect. In cases where partially filled ballast tanks are permitted during ballast voyages, it is imperative to adhere to the guidelines outlined in the loading manual. Sloshing can be described as an interaction between the seawater and the wall structure, which constrains the motion of the liquid.
Strain	S	Strain (stress) following hull loads	Corrosion accelerated by stress is called stress-corrosion cracking. It is generally considered that cracking occurs due to stress induced by various factors [8]. Causes can be flexing of the hull or impact stress due to the previously mentioned sloshing or impact coming from the other side like the cargo or the open sea waves. Another cause can be impact resulting from maneuvering such as mooring or interaction with tugboats. Please note that under “S” mechanical stresses are considered, not stress induced by temperature fluctuations. For the latter, we refer to “D” in the paragraph Dry above. In 2014, Emmanuel Oriaifo compared standards and concluded that they do not adequately replicate cracking failure in water ballast tanks within their service environment [9]. Introducing cracking implies introducing stress.
Cathodic Protection	CP	Sacrificial anodes	Ballast tanks are protected by the use of sacrificial anodes, mostly zinc or aluminum. Cathodic protection is only effective when the tank is filled with seawater. However, the performance of sacrificial anodes degrades with cyclic exposure to both empty atmosphere and immersion in seawater, significantly diminishing the efficacy of cathodic protection [10].

**Table 2:** Correlation scoring of accelerated test protocols.

Test Protocol	Target	Total Wet Time (Immersion and Humid)	Filled Ballast Tank Correlation Score	Empty Ballast Tank Correlation Score	Average Final Total Correlation Score	Remarks
IMO PSPC_15_ dry heat test	dedicated seawater ballast tanks on all types of ships of not less than 500 gross tonnage and for double-side skin spaces arranged in bulk carriers of 150 m in length and upwards	0%	0	1	0.5	
IMO PSPC_15_ condensation chamber test		100%	0	3	1.5	
ISO 20340:2009 (E)	offshore and related structures	64%	0	3	1.5	UV exposure is part of the test but not applicable inside a ballast tank A sub-zero temperature cycle is part of the test but rarely occurs, so not applicable here
ISO 9227:2017(E)	corrosion resistance of metallic materials, with or without permanent or temporary corrosion protection	100%	0	3	1.5	
ASTM D5894-10	cyclic salt fog/UV exposure of painted metal	50%	0	2	1	UV exposure is part of the test but not applicable inside a ballast tank
ASTM B-117	create and maintain the salt spray (fog) test environment	100%	0	3	1.5	
ASTM G 85 annex 5	dilute electrolyte cyclic fog/dry test	50%	0	2	1	
NACE TM0304-2004 seawater immersion resistance test (Section 10)	coating systems for the atmospheric zone and splash zone of an offshore platform	100%	4	0	2	
NACE TM0104-2004 seawater immersion resistance test (Section 8)	offshore platform ballast water tank coating system evaluation	100%	4	0	2	
NACE TM0104-2004 Hot/Wet Cycling Test (Section 13)	offshore platform ballast water tank coating system evaluation for FPSOs only	50%	0	2	1	
NORSOK M-501 refer to ISO 20340	protective coatings to be applied during the construction and installation of offshore installations and associated facilities	64%	0	3	1.5	UV exposure is part of the test but not applicable inside a ballast tank A sub-zero temperature cycle is part of the test but rarely occurs, so not applicable here
NORDTEST Method NT POLY 185	determination of flexibility and fatigue resistance of aged ballast tank coatings	86%	1	2	2	

**Table 3:** Wet time ratio versus corrosion rate of steel, reworked from [19].

Test Procedure	Wet Time Ratio (%)	Corrosion Rate of Steel (g/m^2^/Day)
Cyclic wet (humid and fog) dry	33	27.4–30.9
Cyclic wet (humid and fog) dry	50	76.2–80.1
Cyclic wet (humid and fog) dry	79	108.0–120.0
Continuous fog	100	37.8–40.7

**Table 4:** Temperature settings immersion phases.

Test Procedure	Average Temperature Monitored	Standard Deviation	Coefficient of Variation (σ/Average)
IMMERSION	40.72 °C	0.67 °C	0.33
AMACORT CIFD-01 IMMERSION PHASE	40.17 °C	0.02 °C	0.01

**Table 5:** Rust colors linked to corrosion atmosphere key factors, reworked from [25].

Color	Moisture Content		Water Exposure	Oxygen Content		Comments	Key Factor
	Low	High		Low	High		
Yellow		X			X	appears as yellow run dripping ranges from vivid yellow to dark yellow/orange humid place and the moisture ran over the surface	F
Red			X		X	humid aerated environment uniform corrosion is generally prevalent no rust runs or streaks	F
Brown	X				X	porous rust non-adherent	D
Black			X	X		low or depleted oxygen environment under water conditions	I

**Table 6:** Weight evolution value after exposure of the steel coupons to each of the accelerated corrosion test protocols (immersion, fog/dry and AMACORT CIFD-01) for 2016 h.

Test Procedure	Average Weight Change Straight after the Test (g) (σ = Standard Deviation)	Coefficient of Variation
IMMERSION	−6.42 (σ = 0.04)	0.006
FOG DRY	+37.63 (σ = 1.22)	0.032
AMACORT CIDF-01	+10.03 (σ = 4.04)	0.403

**Table 7:** Corrosion rate for the accelerated corrosion test protocols: immersion, fog/dry and AMACORT CIFD-01.

Test Procedure (Test Time 2016 h or 84 Days or 12 Weeks)	Average Weight Loss in g (σ = Standard Deviation)	Coefficient of Variation of Weight Change (σ/Average)	Loss in g/m^2^/day	mm/Year Thickness Reduction
IMMERSION	6.79 (σ = 0.06)	0.01	5.40	0.25
FOG/DRY	69.69 (σ = 2.64)	0.04	55.38	2.58
AMACORT CIFD-01	83.89 (σ = 4.54)	0.05	66.67	3.10

**Table 8:** Acceleration factor for the accelerated corrosion test protocols: immersion, fog/dry and AMACORT CIFD-01.

Test Procedure (Test Time 2016 h or 84 Days or 12 Weeks)	Average Weight Loss	Weight Loss	Lost Thickness = % Weight Loss of the Initial Thickness 3 mm	Number of Years Aged (1 Year = Loss of 0.4 mm Thickness)	Acceleration Factor Obtained of the Simulated Aging Test Protocol
IMMERSION	6.79 g	1.94%	0.06 mm	55 days	0.65
FOG/DRY	69.69 g	19.98%	0.6 mm	1.5 years (547.5 days)	6.52
AMACORT CIFD-01	83.89 g	24.11%	0.72 mm	1.8 years (657 days)	7.82

## Data Availability

The original contributions presented in the study are included in the article, further inquiries can be directed to the corresponding author.
